# The exit of axons and glial membrane from the developing *Drosophila* retina requires integrins

**DOI:** 10.1186/s13041-021-00888-2

**Published:** 2022-01-03

**Authors:** Qian Ren, Yong Rao

**Affiliations:** 1 McGill Centre for Research in Neuroscience, McGill University Health Centre, 1650 Cedar Avenue, Montreal, QC H3G 1A4 Canada; 2 Department of Neurology and Neurosurgery, McGill University Health Centre, 1650 Cedar Avenue, Montreal, QC H3G 1A4 Canada; 3 Integrated Program in Neuroscience, McGill University Health Centre, 1650 Cedar Avenue, Montreal, QC H3G 1A4 Canada; 4 Centre for Research in Neuroscience, McGill University Health Centre, Room L7-136, 1650 Cedar Avenue, Montreal, QC Canada

## Abstract

Coordinated development of neurons and glia is essential for the establishment of neuronal circuits during embryonic development. In the developing *Drosophila* visual system, photoreceptor (R cell) axons and wrapping glial (WG) membrane extend from the eye disc through the optic stalk into the optic lobe. Extensive studies have identified a number of genes that control the establishment of R-cell axonal projection pattern in the optic lobe. The molecular mechanisms directing the exit of R-cell axons and WG membrane from the eye disc, however, remain unknown. In this study, we show that integrins are required in R cells for the extension of R-cell axons and WG membrane from the eye disc into the optic stalk. Knockdown of integrins in R cells but not WG caused the stalling of both R-cell axons and WG membrane in the eye disc. Interfering with the function of Rhea (i.e. the *Drosophila* ortholog of vertebrate talin and a key player of integrin-mediated adhesion), caused an identical stalling phenotype. These results support a key role for integrins on R-cell axons in directing R-cell axons and WG membrane to exit the eye disc.

## Introduction

The formation of neural networks during embryonic development requires proper neuron-glia interactions. Glial cells produce cues that guide the extension of growing axons. And neurons also produce signals to regulate glial proliferation and differentiation [1, 2]. The mechanisms underlying the coordinated development of neurons and glia for circuit development, however, are still not fully understood.

The establishment of photoreceptor neuron (R cell) to optic lobe connections in the *Drosophila* adult visual system is an excellent model for understanding the mechanisms controlling the coordinated development of neuron and glia for the establishment of neuronal connections. R-cell differentiation in the eye-imaginal disc begins at the third-instar larval stage, and progresses in a posterior-to-anterior direction across the eye disc [3, 4]. Within each ommatidium, R8 differentiates first, followed by R2/5, R3/4, R1/6, and R7. Differentiating R cells project axons that migrate towards the posterior end of the eye disc, where they exit the eye disc and into the optic stalk, a tubular structure connecting the eye disc to the optic lobe. After exiting the optic stalk, R-cell axons project into first (i.e. lamina) and second (i.e. medulla) layers of the optic lobe, and establish a precise retinotopic map [5].

Wrapping glia (WG), a subtype of sub-retinal glia, play an important role in the establishment of retinotopic map [6, 7]. WG are derived from perineurial glia (PG) in the optic stalk. At the third-instar larval stage, PG migrate from the optic stalk into the sub-retinal region of the eye disc [8]. After exposure to FGF8-like ligand Thisbe from differentiating R cells, PG differentiate into WG [9]. R-cell axons are temporally enwrapped by WG membrane, which extends from the eye disc through the optic stalk into the distal region of the lamina [6, 10, 11]. Our previous studies show that the ensheathment of R-cell axons by WG requires two cell-surface adhesion molecules Turtle (Tutl) and Borderless (Bdl) [10, 11], both of which belong to the conserved IgSF9 subfamily of the immunoglobulin superfamily [12]. We show that the interactions between Tutl on R-cell axons and Bdl on WG are required for WG extension and the ensheathment of R-cell axons [10, 11]. The extension of WG membrane from the eye disc into the optic lobe is required for the proper organization of R-cell axons within the optic stalk [10, 11], the topographic projection of R-cell axons in the optic lobe [6], and the differentiation of lamina neurons in the optic lobe [7].

The molecular mechanisms that direct R-cell axons and WG membrane to exit the eye disc remain unknown. Several studies suggest that sub-retinal glia may provide cues at the posterior end of the eye disc that allow the exit of R-cell axons from the eye disc [13–15]. The molecular identity of glial-derived cues and their receptors on R-cell axons, however, remains elusive. While the temporal ensheathment of R-cell axons by WG during development leads to speculation that R-cell axons promote the extension of WG membrane, it remains unclear if the extension of WG membrane from the eye disc into the optic stalk is dependent on R-cell axons. In this study, we show that integrins are required in R cells but not WG for the exit of both R-cell axons and WG membrane from the eye disc.

## Results

### Knockdown of *mys* in both eye-disc epithelium and WG prevented the exit of R-cell axons and WG membrane from the eye disc

In our previous study [16], we performed a RNA interference (RNAi) screen to search for cell-surface receptors and secreted factors that regulate the coordinated development of R cells and WG in the developing *Drosophila* visual system. We found that when a *UAS*-*RNAi* transgene (i.e. *UAS*-*mys-RNAi-HMS00043*) was expressed in both WG and eye-disc epithelium consisting of differentiating R cells and accessory cells, R-cell axons and WG membrane stalled in the eye disc [16]. Since *UAS*-*mys-RNAi-HMS00043* targets the *myospheroid (mys)* gene that encodes for a beta integrin subunit (βPS), this result suggests a role for Mys in mediating the exit of R-cell axons and WG membrane from the eye disc. To further test this, we examined another independent RNAi line (i.e. *UAS*-*mys-RNAi-JF02819*) targeting a different region of *mys*.

**Fig. 1:**
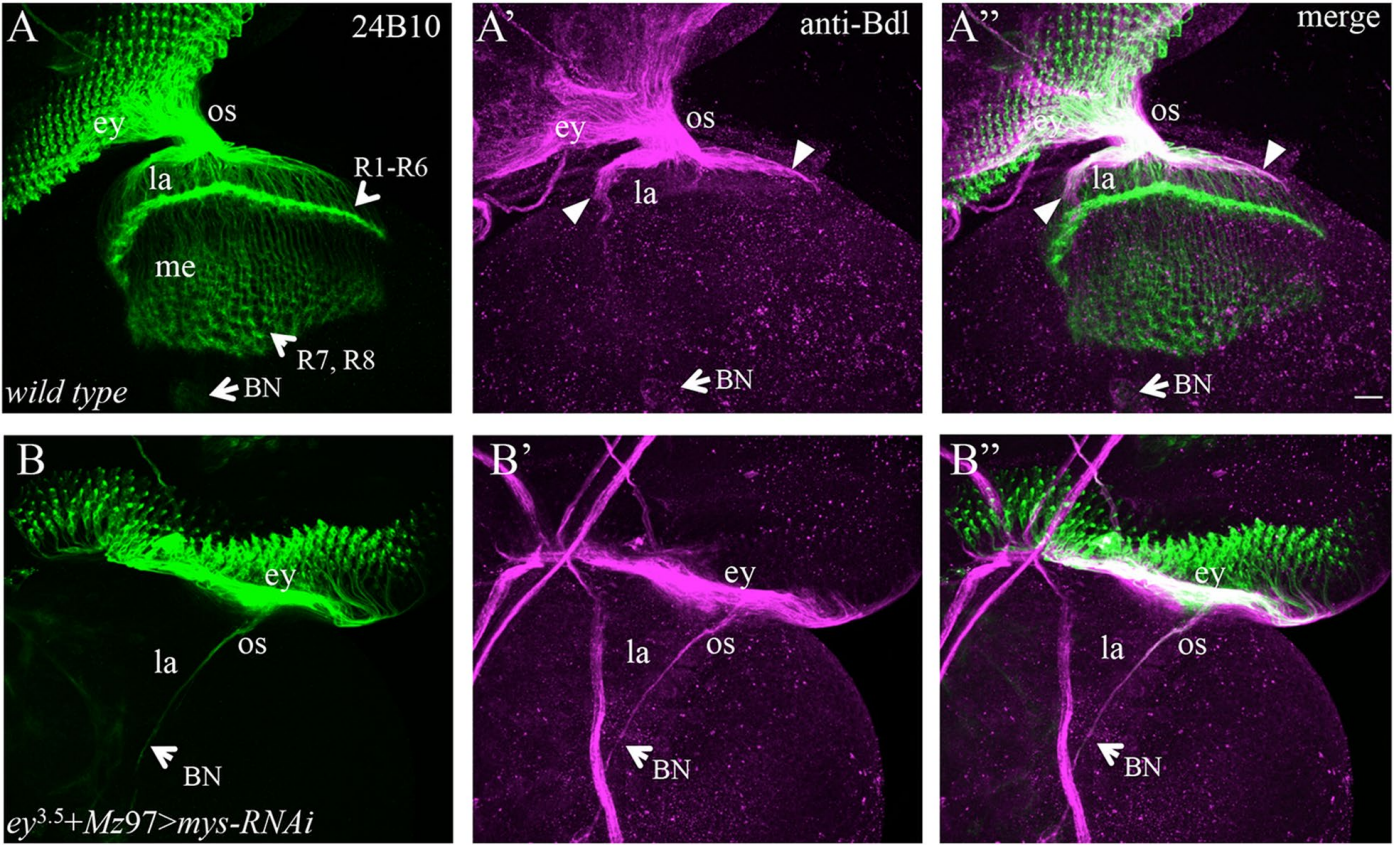
Knocking down *mys* in both WG and eye-disc epithelium caused a failure of R-cell axons and WG membrane to exit the eye disc. 3rd-instar larval eye–brain complexes were double-stained with MAb24B10 (green) and anti-Bdl (magenta). MAb24B10 (green) labels all R-cells, and anti-Bdl (magenta) labels WG. **A**–**Aʺ** The pattern of R-cell axonal projection and WG membrane extension in wild type. **A** MAb24B10 staining. R-cell axons migrate towards the posterior end of the eye disc (ey) where they converge and enter the optic stalk (os). After exiting the optic stalk, R1–R6 axons terminate at the intermediate target region in the lamina (la), and R7 and R8 axons pass through the lamina into the deeper medulla layer (me). **Aʹ** The same section stained with anti-Bdl to visualize WG. WG membrane extends from the eye disc through the optic stalk into the distal region of the lamina (arrowheads). MAb24B10 and anti-Bdl staining also visualized Bolwig’s Nerve (BN in **A** and **Aʹ**) that projects from Bolwig’s organ in the larval anterior region into the optic lobe long before the birth of R cells and WG. **Aʺ** The section visualized with both MAb24B10 and anti-Bdl staining. Note that WG membrane associates with R-cell axons in the lamina (arrowheads). **B**–**Bʺ** A third-instar eye–brain complex in which *UAS-mys-RNAi-JF02819* was simultaneously expressed in both eye-disc epithelium and WG under control of the eye-specific *ey*^3.5^-GAL4 and WG-specific *Mz97*-GAL4 drivers. *mys* knockdown caused the stalling of R-cell axons (**B** and **Bʺ**) and WG membrane (**Bʹ** and **Bʺ**) in the eye disc. Note *mys* knockdown did not affect the projection pattern of pre-existing Bolwig’s Nerve (BN). Scale bar: 20 μm

*UAS*-*mys-RNAi-JF02819* was simultaneously expressed in both R cells and WG under control of the eye-specific *ey*^3.5^-GAL4 and WG-specific *Mz97*-GAL4. The R-cell axonal projection pattern was visualized using MAb 24B10, which recognizes the R-cell-specific cell adhesion molecule Chaoptin [17]. The extension of WG membrane was visualized with antibodies against Bdl that is specifically expressed on WG membrane at the third-instar larval stage [11]. In wild type (Fig. 1A, Aʺ), R-cell axons migrate posteriorly and converge at the most posterior end where they leave the eye disc and into the optic stalk. After exiting the optic stalk, R1–R6 axons terminate at the intermediate target region in the lamina, and R7 and R8 axons pass through the lamina into the deeper medulla. The extension of WG membrane is closely associated with R-cell axons from the eye disc through the optic stalk into the distal region of the lamina, where WG membrane ceases extension and dissociates from R-cell axons (Fig. 1Aʹ, Aʺ).

In most animals expressing UAS-*mys-RNAi-JF02819* in both eye-disc epithelium and WG (Fig. 1B–Bʺ, Table 1), R-cell axons and WG membrane extended normally towards the posterior end of the eye disc, but failed to exit the eye disc or stalled in the optic stalk. These phenotypes were identical to that in animals expressing *UAS*-*mys-RNAi-HMS00043* in both eye-disc epithelium and WG reported in our previous study [16]*.*

**Table 1:** Summary of R-cell axon and WG membrane stalling phenotypes observed in mutants defective in the integrin pathway

Genotype	Penetrance of the stalling phenotype	Number of individuals in which most R-cell axons and WG stalled in the eye disc	Number of individuals in which many R-cell axons and WG stalled in the optic stalk
*ey*^3.5^ + *Mz*97 > *mys-RNAi-JF02819*	9/15	8	1
*ey*^3.5^ > *mys-RNAi-HMS00043*	10/13	9	1
*C155* > *mys-RNAi-HMS00043*	9/16	3	6
*ey*^3.5^ + *Mz*97 > *scb-RNAi-JF02696*	6/10	5	1
*ey*^3.5^ > *scb-RNAi-JF02696*	12/14	9	3
*ey*^3.5^ > *rhea-RNAi-HMS00799*	11/12	9	2
*ey*^3.5^ > *rhea-RNAi-HMS00856*	10/12	9	1
*rhea*^1^ eye-specific mosaic	15/25	15	0

### Knockdown of *mys* in R cells but not WG caused the stalling of R-cell axons and WG membrane in the eye disc

The above results raise three possibilities for the role of *mys* in the exit of R-cell axons and WG membrane: (1) *mys* is only required in R cells; (2) *mys* is only required in WG; and (3) *mys* is required in both R cells and WG. To distinguish among these possibilities, we performed knockdown experiments by expressing *UAS-mys*-*RNAi* transgene under control of the eye-specific *ey*^3.5^-GAL4 only or the WG-specific *Mz97*-GAL4 only. *ey*^3.5^-GAL4 drives the expression of *UAS-mys-RNAi* transgene in eye-disc epithelium consisting of both R cells and accessory cells, but not in WG or other sub-retinal glia. Whereas *Mz*97-GAL4 drivers the expression of *UAS-mys-RNAi* transgene specifically in WG [14].

**Fig. 2:**
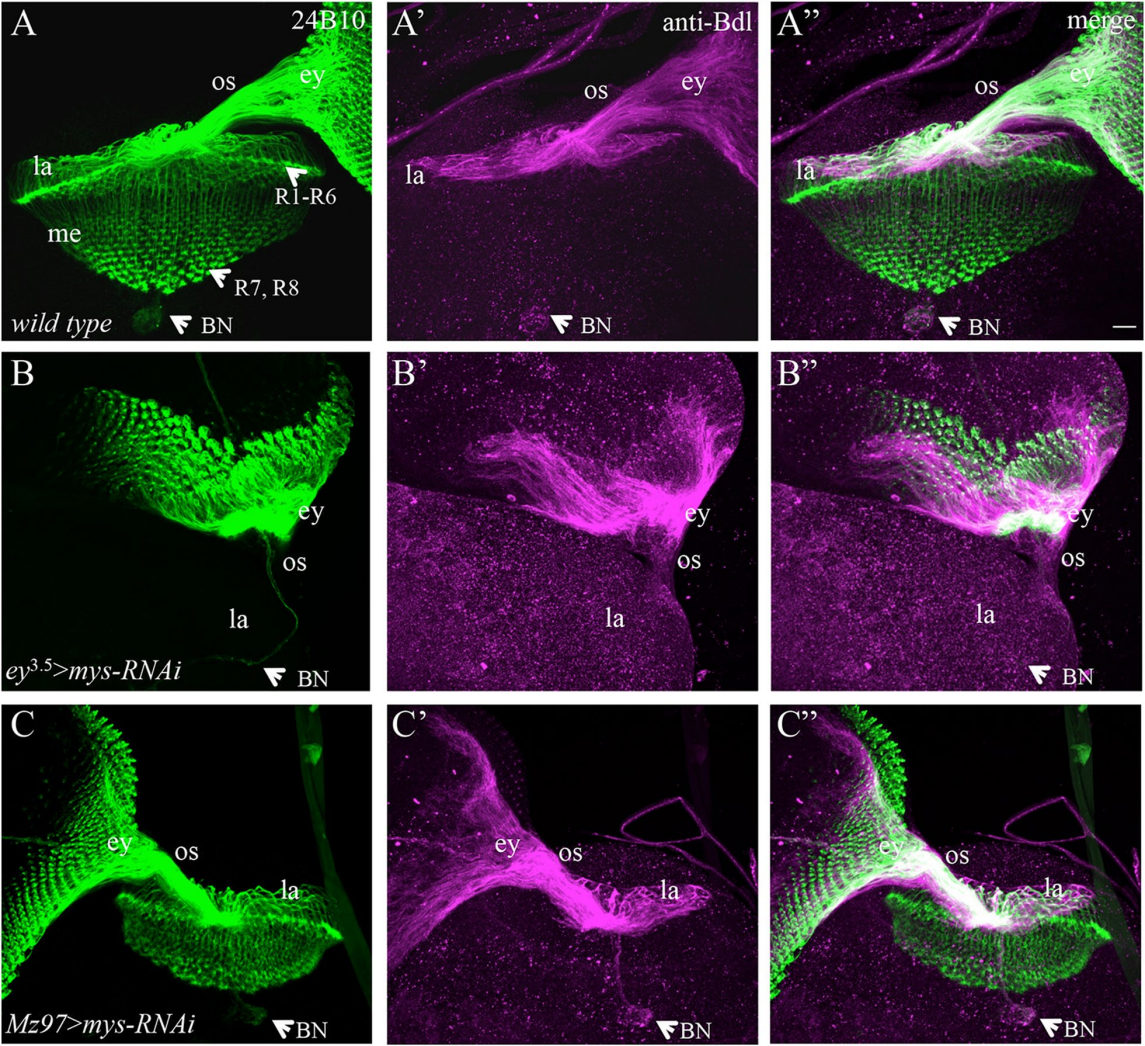
Knockdown of *mys* in eye-disc epithelium but not WG prevented the exit of R-cell axons and WG membrane. 3rd-instar larval eye–brain complexes were double-stained with MAb24B10 (green) and anti-Bdl (magenta). **A**–**Aʺ** Wild type. **B**–**Bʺ** Knocking down *mys* in eye-disc epithelium only by expressing *UAS-mys-RNAi-HMS00043* under control of *ey*^3.5^-GAL4 (i.e. *ey*^3.5^ > *mys-RNAi*) caused a stalling phenotype identical to that in animals with *mys* knockdown in both eye-disc epithelium and WG (Fig. 1B–Bʺ). **C**–**Cʺ** The pattern of R-cell axonal projection and WG membrane extension appeared normal in animals in which *mys* was only knocked down in WG by expressing *UAS-mys-RNAi-HMS00043* under control of *Mz97*-GAL4 (i.e. Mz97 > *mys-RNAi*) (n = 16). Scale bar: 20 µm

Knockdown of *mys* under control of *ey*^3.5^*-*GAL4 caused a stalling phenotype (Fig. 2B–Bʺ) identical to that in animals in which *mys* was knocked down in both eye-disc epithelium and WG (Fig. 1B–Bʺ, Table 1). In contrast, WG-specific knockdown of *mys* under control of *Mz97-*GAL4 did not affect the exit of R-cell axons or WG membrane (Fig. 2C–Cʺ). These results indicate that *mys* is required in eye-disc epithelium but not WG for the exit of both R-cell axons and WG membrane from the eye disc.

**Fig. 3:**
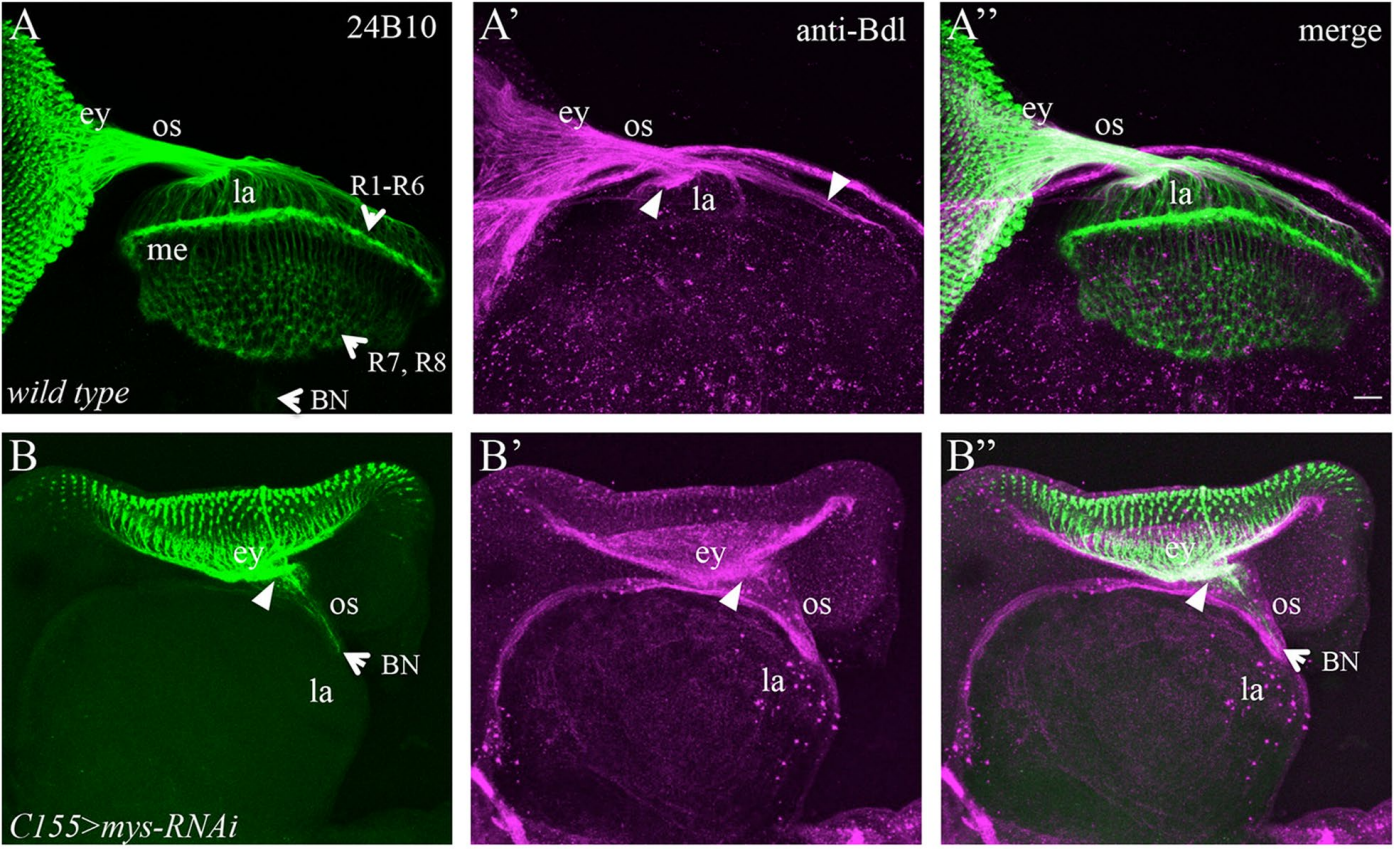
Neuronal-specific knockdown of *mys* caused a similar stalling phenotype. 3rd-instar larval eye–brain complexes were double-stained with MAb24B10 (green) and anti-Bdl (magenta). **A**–**Aʺ** Wild type. Note that only Bdl-positive processes (arrowheads) that were associated with R-cell axons in the lamina were WG membrane. **B**–**Bʺ** *mys* was specifically knocked down in all differentiating R cells (the only neuronal cell types in the eye disc) by expressing *UAS-mys-RNAi-HMS00043* under control of the neuronal-specific *C155*-GAL4 (i.e. *C155* > *mys-RNAi*). Most R-cell axons and WG membrane stalled at the posterior end of the eye disc (arrowheads). Scale bar: 20 µm

Since eye-disc epithelium consists of both R cells and accessory cells, the above phenotypes may reflect a role for *mys* in R cells and/or accessory cells in mediating the exit of R-cell axons and WG. To further determine cell-type-specific requirements of *mys*, we performed knockdown experiments using the pan-neuronal-specific driver *C155-*GAL4, which turned on the expression of RNAi transgenes in R cells but not accessory cells in eye-disc epithelium. Consistently, we found that knocking down *mys* under control of *C155-*GAL4 caused the stalling of R-cell axons and WG membrane in the eye disc (Fig. 3B–Bʺ, Table 1). Since R cells are the only neuronal cell types in the eye disc, this result indicates that *mys* is required in R cells for the exit of R-cell axons and WG membrane from the eye disc.

### Mys is expressed in R-cell axons

**Fig. 4:**
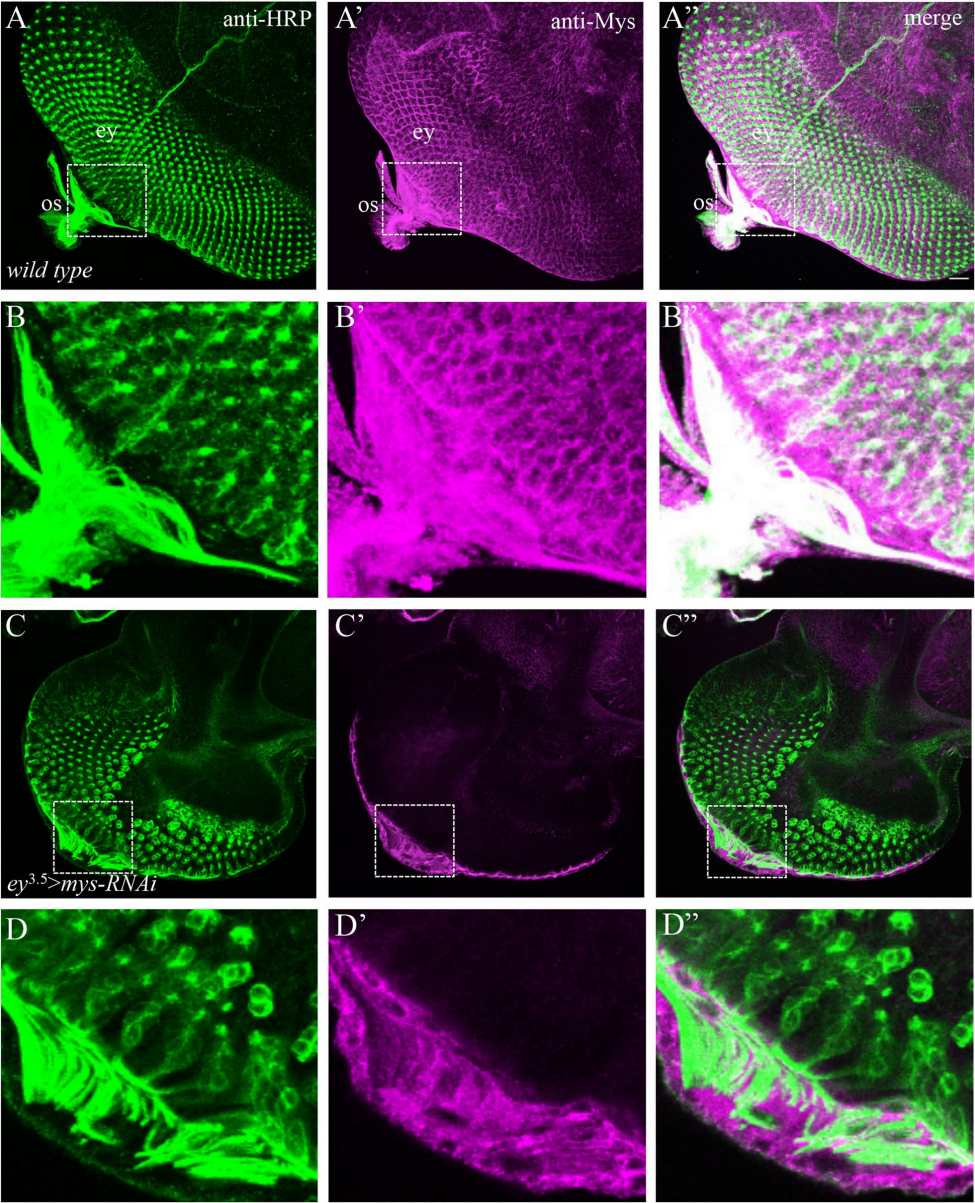
Mys is expressed in R-cell axons. 3rd-instar larval eye discs were double-stained with anti-HRP (green) and anti-Mys antibodies (magenta). **A** Wild type, anti-HRP staining visualized R-cell soma and axons. **Aʹ** The same section stained with anti-Mys antibody. **Aʺ** The section visualized with both anti-HRP and anti-Mys staining. Mys appeared to be expressed broadly in the eye disc. Strong Mys expression was detected in R-cell axons that migrate from the most posterior end of the eye disc into the optic stalk. **B**–**Bʺ** Enlarged view of the boxed area in **A**–**Aʺ**. Note the strong Mys staining in R-cell axons. **C**–**Cʺ** When *mys* was knocked down in eye-disc epithelium but not WG by expressing *UAS-mys-RNAi-HMS00043* under control of *ey*^3.5^-GAL4, the intensity of Mys staining was greatly reduced in both R-cell soma and accessory cells in the eye disc, confirming that Mys is expressed in both R cells and accessory cells. The staining in R-cell axons was also significantly reduced. **D**–**Dʺ** Enlarged view of the boxed area in **C**–**Cʺ**. The intensity of Mys staining in R-cell axons was greatly reduced. Note anti-Mys staining at the most posterior region of the eye disc in knockdown animals were mostly associated with sub-retinal glia but not R-cell axons, which is consistent with that expression of *UAS-mys-RNAi-HMS00043* under control of *ey*^3.5^-GAL4 knocked down *mys* in eye-disc epithelium but not sub-retinal glia. Scale bar: 20 µm

The above results suggest a role for Mys in R cells for the exit of R-cell axons and WG membrane from the eye disc. To further address this, we examined the expression pattern of Mys in the eye disc by staining third-instar eye disc with a mouse anti-Mys monoclonal antibody [18]. Consistent with a previous report [19], we found that Mys was broadly expressed in the eye disc (Fig. 4Aʹ, Aʺ). At the posterior end of the eye disc and the optic stalk, strong Mys staining was detected in R-cell axons (Fig. 4Bʹ, Bʺ). The intensity of anti-Mys staining was greatly reduced when *mys* was specifically knocked down in the eye disc (Fig. 4Cʹ, Cʺ, Dʹ and Dʺ), supporting the specificity of this antibody.

### Knockdown of *mys* in R-cell axons did not affect the migration of glial cells from the optic stalk into the eye disc

**Fig. 5:**
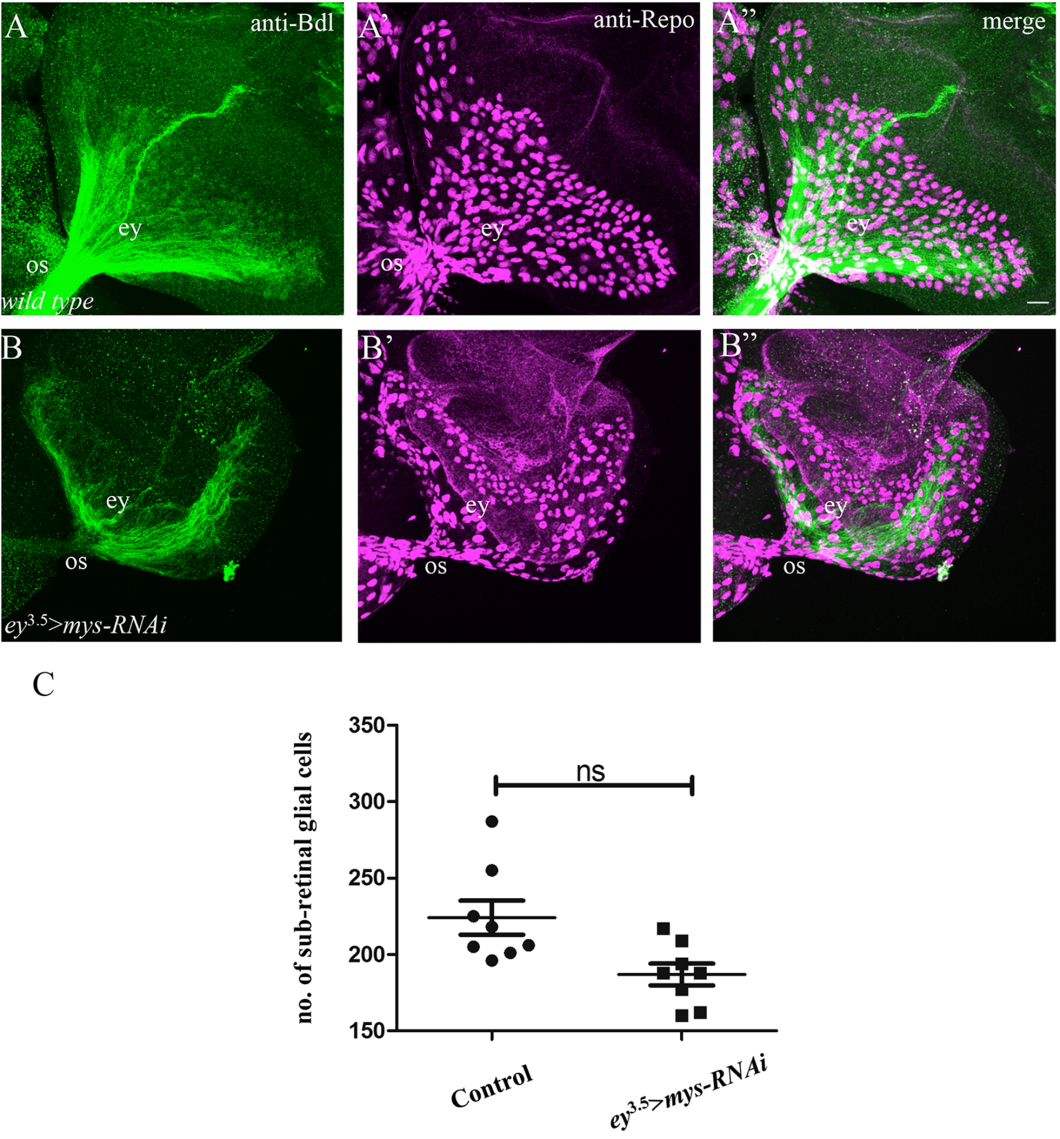
Knocking down *mys* in eye-disc epithelium did not affect the migration of glia from the optic stalk into the eye disc. 3rd-instar larval eye–brain complexes were double-stained with anti-Bdl (green) and anti-Repo (magenta). Anti-Bdl and anti-Repo label WG membrane and all glial nuclei, respectively. **A**–**Aʺ** In wild-type, glial cells in the optic stalk migrate into the sub-retinal region of the eye disc. **B**–**Bʺ** When *mys* was knocked down in eye-disc epithelium by expressing *UAS-mys-RNAi-HMS00043* under control of *ey*^3.5^-GAL4, glial cells still migrated into the sub-retinal region of the eye disc. **C** The number of glial cells in the eye disc was counted. The number of sub-retinal glial cells in *mys* knockdown eye disc is not significantly (ns, p > 0.05) different from that in wild type. Scale bar: 20 µm

Previous studies suggest that the migration of glial cells from the optic stalk into the eye disc is required for the exit of R-cell axons from the eye disc [13–15]. This raises the possibility that the stalling defects in animals in which *mys* was knocked down in R cells (Figs. 2 and 3), were due to a failure of glial migration into the eye disc. To test this, we examined if knocking down *mys* in R cells affects glial migration. Glial cells in the sub-retinal region of the eye disc were stained with a mouse monoclonal antibody that recognizes the Reversed polarity protein (Repo) expressed in the nuclei of all glia [20]. Compared to that in wild type (Fig. 5A–Aʺ, C), the number of sub-retinal glial cells was not significantly reduced in knockdown animals (Fig. 5B–Bʺ, C). In *mys* knockdown eye discs showing stalling phenotypes, the number of sub-retinal glia varied from 160 to 229. Given that the presence of 3 to 20 sub-retinal glia in the eye disc was shown to be sufficient for the exit of R-cell axons from the eye disc into the optic stalk [13], this result argues against that Mys mediates the exit of R-cell axons by promoting the migration of glial cells from the optic stalk into the eye disc.

### Knockdown of *scab* (*scb*) encoding for αPS3 integrin subunit caused a *mys*-like R-cell axon and WG stalling phenotype

The integrin receptor is a heterodimer consisting of α and β subunits. In *Drosophila*, Mys (βPS) functions by forming a heterodimer with one of five α-integrin subunits [21]. Among the five α-integrin subunits, Inflated (If) (i.e. αPS2) and Scb (i.e. αPS3) are reported to be expressed in the eye disc and optic stalk [15]. However, knockdown of *if* in both eye-disc epithelium and WG did not affect the exit of R-cell axons or WG membrane from the eye disc [16], arguing against that Mys functions together with If in R-cell axons. To determine the role of *scb* in R-cell axons, we examined the effects of *scb* knockdown on the extension of R-cell axons and WG.

**Fig. 6:**
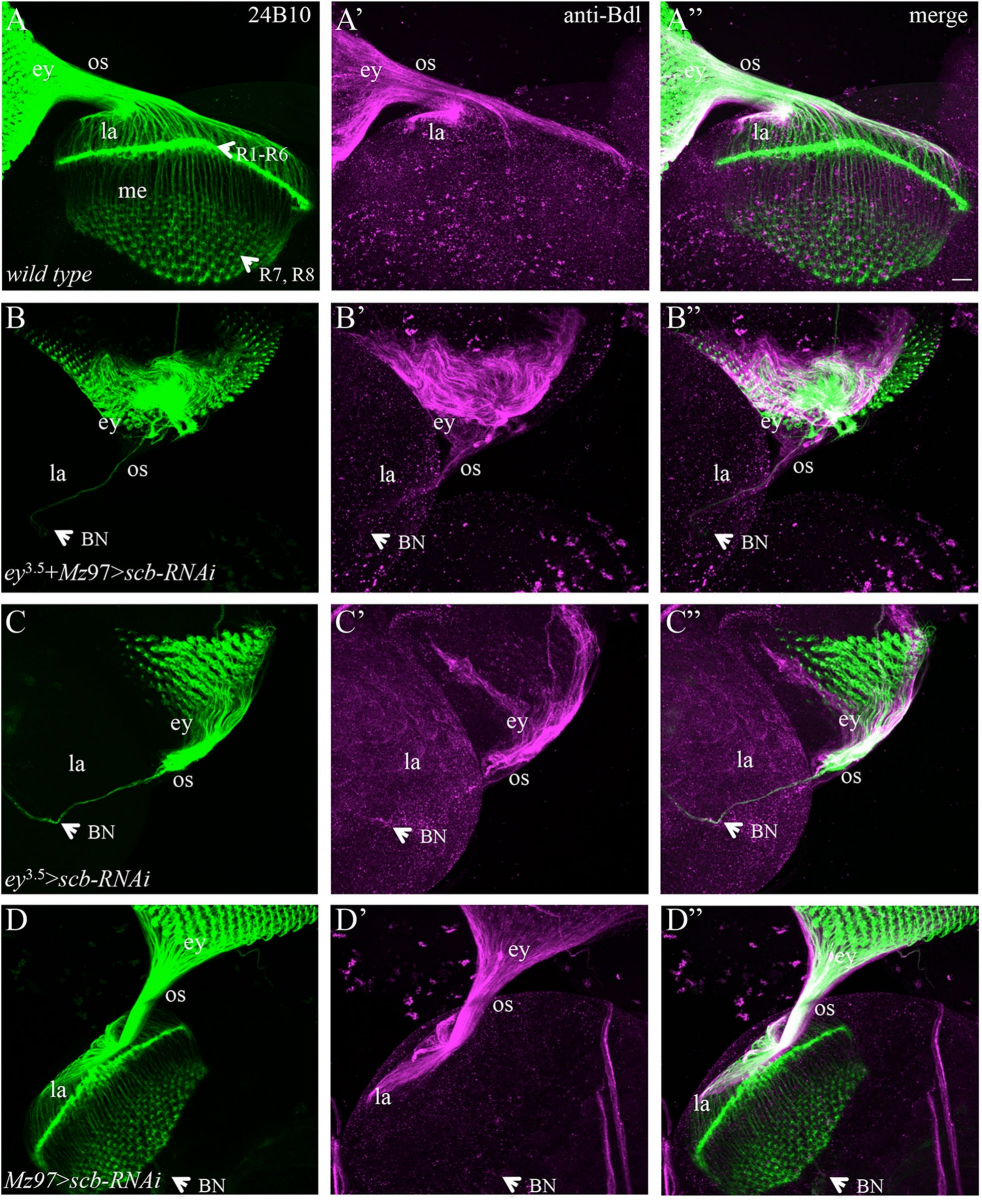
Knockdown of *scb* in eye-disc epithelium but not WG prevented the exit of R-cell axons and WG membrane. 3rd-instar larval eye–brain complexes were double-stained with MAb24B10 (green) and anti-Bdl (magenta). **A**–**Aʺ** Wild type. **B**–**Bʺ** when *scb* was knocked down in both eye-disc epithelium and WG by expressing *UAS-scb-RNAi-JF02696* under control of *ey*^3.5^-GAL4 and *Mz97*-GAL4, most R-cell axons and WG membrane stalled in the eye disc. **C**–**Cʺ** In an individual in which *scb* was knocked down only in eye-disc epithelium by expressing *UAS-scb-RNAi-JF02696* under control of *ey*^3.5^-GAL4, some R-cell axons and WG membrane exited the eye disc and stalled in the optic stalk. **D**–**Dʺ** WG-specific knockdown of *scb* by expressing *UAS-scb-RNAi-JF02696* under control of *Mz97*-GAL4 did not affect R-cell axonal projection or WG membrane extension (n = 13). Scale bar: 20 µm

Like *mys* knockdown, knockdown of *scb* in both eye-disc epithelium and WG caused the stalling of R-cell axons and WG membrane in the eye disc or in the optic stalk (Fig. 6B–Bʺ, Table 1). Similar defects were observed when *scb* was specifically knocked down in eye-disc epithelium (Fig. 6C–Cʺ, Table 1), while no defect was observed when *scb* was knocked down in WG only (Fig. 6D–Dʺ). These results suggest strongly that Mys and Scb function together in R-cell axons to mediate the exit of R-cell axons and WG membrane.

### Interfering with the function of Rhea (the fly ortholog of talin) in eye-disc epithelium prevented the exit of R-cell axons and WG membrane from the eye disc

The requirements of Mys and Scb for the exit of R-cell axons and WG membrane support a key role for integrin-mediated adhesion in this process. To further address this, we examined if interfering with the function of Rhea [22], the fly ortholog of talin and a key player of the integrin-mediated adhesion [23], causes phenotypes similar to that observed in *mys* or *scb* knockdown animals.

**Fig. 7:**
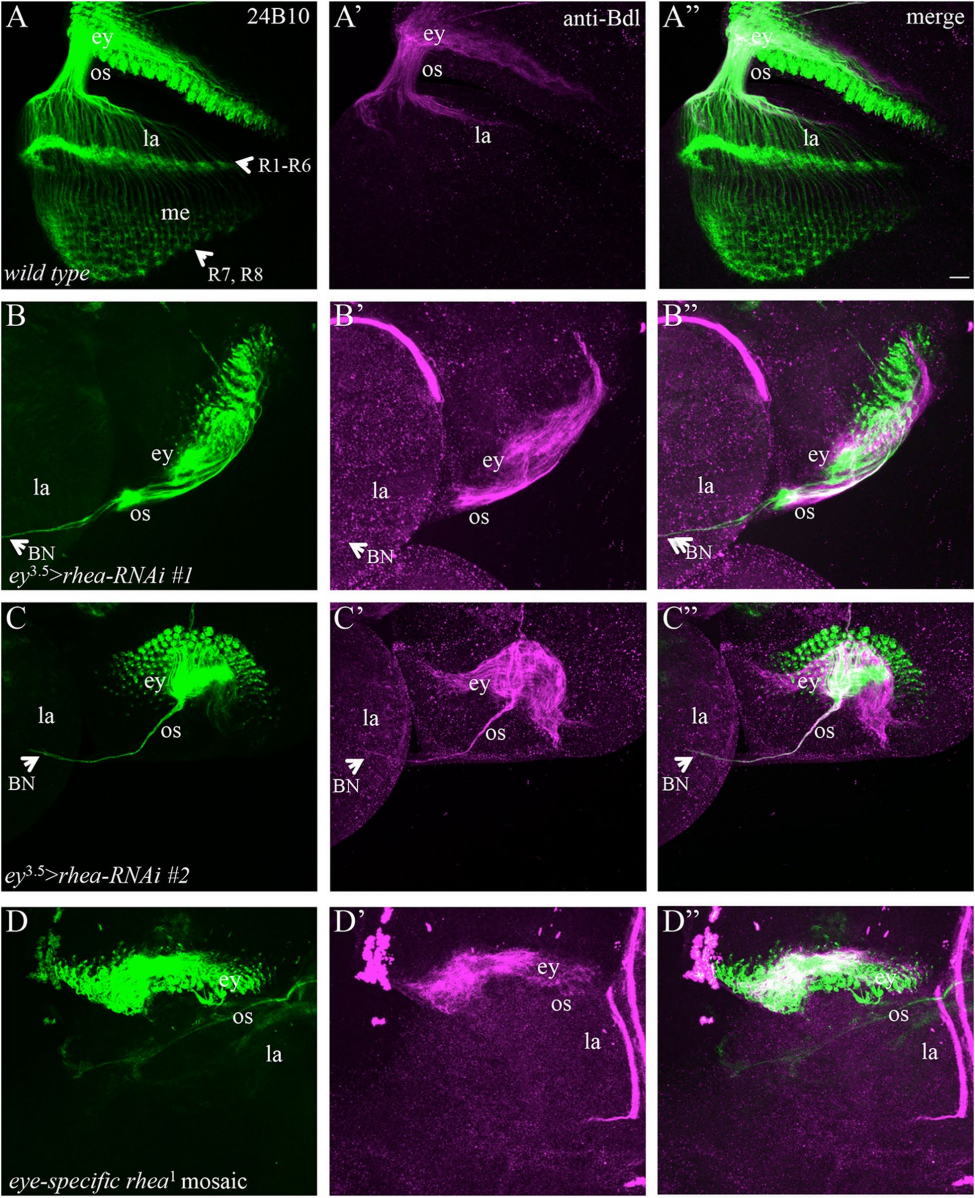
Interfering with the function of Rhea in eye-disc epithelium but not WG also caused the stalling of R-cell axons and WG membrane in the eye disc. 3rd-instar larval eye–brain complexes were double-stained with MAb24B10 (green) and anti-Bdl (magenta). **A**–**Aʺ** Wild type. **B**–**Bʺ** In an individual in which *rhea* was knocked down in the eye disc by expressing *UAS-rhea-RNAi-HMS00799* (i.e. *UAS-rhea-RNAi #1*) under control of *ey*^3.5^-GAL4, the stalling of R-cell axons and WG membrane in the eye disc and optic stalk was observed. **C**–**Cʺ** In an individual in which *rhea* was knocked down only in eye-disc epithelium by expressing another independent *UAS-rhea-RNAi* line (i.e. *UAS-rhea-RNAi-HMS00856* or *UAS-rhea-RNAi #2*) under control of *ey*^3.5^-GAL4, most R-cell axons and WG membrane stalled in the eye disc. **D**–**Dʺ** In many eye-specific mosaic animals in which large clones of homozygous *rhea*^1^ mutant eye tissues were generated, R-cell axons and WG membrane failed to exit the eye disc. Scale bar: 20 µm

Like that in *mys* and *scb* knockdown animals, R-cell axons and WG membrane failed to exit the eye disc or stalled in the optic stalk when *rhea* was knocked down in eye-disc epithelium but not WG (Fig. 7B–Bʺ and C–Cʺ, Table 1). In contrast, no phenotype was observed when *rhea* was knocked down in WG only (0 out of 11 animals examined). Similar results were obtained using two independent *rhea-RNAi* lines (Fig. 7B–Bʺ and C–Cʺ).

We also performed genetic mosaic analysis to examine if the removal of *rhea* specifically in eye-disc epithelium but not sub-retinal glia, disrupts R-cell axonal projections and WG extension. The *eyFLP-FRT* system was used to generate large clones of mutant eye tissue homozygous for *rhea*^1^, a strong *rhea* loss-of-function mutant allele [22]. Consistent with the results from eye-specific knockdown experiments, mosaic animals carrying large clones of homozygous *rhea*^1^ eye tissues also displayed defects (Fig. 7D–Dʺ) that were identical to that in eye-specific *mys* or *rhea* knockdown animals. These results support an essential role for integrin-mediated adhesion in mediating the exit of R-cell axons and WG membrane from the eye disc.

## Discussion

In this study, we provide several lines of evidence that supports a specific role for integrins on R-cell axons to direct the exit of R-cell axons and WG membrane from the eye disc. First, knockdown of βPS integrin subunit Mys or αPS3 integrin subunit Scb in eye-disc epithelium but not WG caused the stalling of both R-cell axons and WG membrane in the eye disc. Second, knockdown of Mys specifically in R cells in eye-disc epithelium also prevented the exit of R-cell axons and WG membrane from the eye disc. Third, Mys is strongly expressed on R-cell axons at the posterior end of the eye disc where R-cell axons converge and extend into the optic stalk. And fourth, interfering with the function of the fly talin (i.e. Rhea) in eye-disc epithelium but not WG prevented the extension of R-cell axons and WG membrane from the eye disc into the optic stalk.

To our knowledge, this study identifies for the first time a cell-surface receptor on R-cell axons that plays an essential and specific role in mediating the exit of R-cell axons and WG membrane from the eye disc. In several previous studies [24–26], forward genetic screens were performed to search for key players that control R-cell axonal projections. Although these studies led to the identification of a number of important genes required for R-cell axonal guidance and layer-specific target selection in the optic lobe, no R-cell surface receptor that specifically controls the exit of R-cell axons from the eye disc was uncovered from these genetic screens.

It is reported that in the developing mouse retina, netrin-1 from neuroepithelial cells interacts with its receptor DCC on retinal ganglion axons to mediate the exit of retinal ganglion axons from the optic disc into the optic nerve [27]. However, the fly orthologs of netrins (i.e. Netrin A and B) and DCC (i.e. Frazzled) are not required for the exit of R-cell axons from the eye disc into the optic stalk [28]. We speculate that like the action of DCC on retinal ganglion axons, integrins on R-cell axons may recognize specific cues at the posterior end of the eye disc for the extension of R-cell axons and WG membrane into the optic stalk. And sub-retinal glia at the posterior end of the eye disc may present such cues that are recognized by integrins on R-cell axons. Consistent with this notion, it is reported that blocking the migration of glial cells into the sub-retinal region of the eye disc caused the failure of R-cell axons to exit the eye disc [13, 15]. Integrins on R-cell axons may interact with certain cell-surface receptors on sub-retinal glia, which may serve as permissive substrates for the extension of R-cell axons. Alternatively or additionally, extracellular matrix (ECM) proteins associated with sub-retinal glia may be recognized by integrins on R-cell axons to promote the exit of R-cell axons. It is reported that glia-specific knockdown of integrins could cause the stalling of R-cell axons in the eye disc without affecting the migration of glial cells into the eye disc [29]. One likely explanation is that integrins on sub-retinal glia may regulate the organization of ECM proteins, and thus allow the ECM proteins to be properly recognized by integrins on R-cell axons to direct the exit of R-cell axons from the eye disc.

Our phenotypic analysis of mutants defective in the integrin pathway also showed that some R-cell axons and WG membrane were able to exit the eye disc and then stalled within the optic stalk (Table 1, Figs. 6C–Cʺ and 7B–Bʺ). One likely explanation is that knockdown efficiency may vary between animals, which contributed to phenotypic variability. That R-cell axons stalled in the optic stalk in some knockdown animals, further suggests that integrins on R-cell axons may also play a role in promoting the migration of R-cell axons through the optic stalk. Like that at the posterior end of the eye disc, cell-surface receptors and/or ECM proteins in the optic stalk may be recognized by integrins, and thus serve as permissive substrates for the migration of R-cell axons through the optic stalk.

Fly integrins are highly homologous to their mammalian counterparts [30]. The closest homologs of Mys and Scb in mammals are β1 integrin and α4/9 integrin [30], respectively. In mammals, β1 integrin and α4/9 integrin have been shown to be involved in mediating axonal projections [31–34]. For instance, it is reported that Semaphorin 7A binds to β1 integrin to promote axon growth in the mouse embryo [31], and α4 integrin interacts with VCAM-1 to mediate the innervation of the heart by sympathetic axons in rats [32]. Whether β1 integrin and α4/9 integrin are also required for the exit of retinal ganglion axons from the optic disk in mammals, however, remains unclear. Interestingly, it is reported that the ECM protein laminin-1 interacts with netrin-1 to mediate the exit of retinal ganglion axons from the optic disk in Xenopus [35]. Given that β1 integrins are well-known laminin-1 receptors [36, 37], it is highly likely that like the action of Mys in the fly eye disc, mammalian β1 integrins may also play a role in the exit of retinal ganglion axons from the optic disk.

Our results show that integrins are necessary for the exit of R-cell axons from the eye disc, but not required for the extension of R-cell axons towards the posterior end of the eye disc. The mechanisms that direct the posterior migration of R-cell axons in the eye disc remain unknown. Since R-cell axons migrate on the apical surface of carpet glia in the eye disc [38, 39], it is possible that the interactions between R-cell axons and carpet glia may be mediated by different cell surface receptors, which direct the posterior extension of R-cell axons independent of integrins. Alternatively or additionally, R-cell axons may be directed towards the posterior end of the eye disc by some unknown chemoattractants.

Our results suggest strongly that the extension of WG membrane from the eye disc through the optic stalk into the optic lobe is dependent on R-cell axons. In mutants defective in integrin signalling in R-cell axons, the extension of both R-cell axons and WG from the eye disc into the optic stalk was disrupted. We never observed an individual in which WG membrane still extended into the optic stalk when R-cell axons stalled in the eye disc. Cell-surface molecules on R-cell axons may serve as permissive substrates for the extension of WG membrane. In our previous studies [10, 11], we show that the recognition between R-cell axons and WG mediated by Tutl on R-cell axons and its binding partner Bdl on WG membrane, is required for the extension of WG membrane in the optic lobe. However, loss of *tutl* or *bdl* did not prevent the exit of WG membrane from the eye disc [10, 11], suggesting the involvement of other cell-surface players. Future studies are needed to identify additional key players that mediate the recognition between R-cell axons and WG for the extension of WG membrane from the eye disc into the optic stalk.

## Conclusion

The present study supports that the integrin receptor consisting of the βPS integrin subunit Mys and the αPS3 integrin subunit Scb on R-cell axons, mediates the exit of R-cell axons from the eye disc into the optic stalk. Integrins on R-cell axons may recognize specific cues produced by sub-retinal glia at the posterior end of the eye disc. That Mys, Scb and the fly talin (i.e. Rhea) are all required for the exit of R-cell axons, supports an essential role for integrin-mediated adhesion in directing the extension of R-cell axons from the eye disc into the optic stalk. This study also suggests strongly that the extension of WG membrane from the eye disc into the optic stalk is dependent on R-cell axons, which may express some cell-surface receptors that serve as permissive substrates for the extension of WG membrane.

## Materials and methods

### Genetics

Fly stocks are reared at 25 °C with 50% humidity and 12/12 h light–dark cycle. Following stocks were obtained from Bloomington *Drosophila* Stock Center (BDSC): *UAS-mys-RNAi-JF02819* (BDSC#27735), *UAS-mys-RNAi-HMS00043* (BDSC#33642), *UAS-scb-RNAi-JF02696* (BDSC#27545), *UAS-rhea-RNAi-HMS00799* (BDSC#32999), *UAS-rhea-RNAi-HMS00856* (BDSC#33913), *yw; P{white-un1}70C rhea*^1^* P{neoFRT}80B/TM6B, P{iab-2(1.7)lacZ}6B, Tb *(BDSC#2296), and *yw; P{GAL4-ey.H}3–8, P{UAS-FLP.D}JD1; P{GMR-hid}SS3, l(3)CL-L1, P{Car20y}73C P{neoFRT}80B/TM2* (BDSC#43658). *Mz97*-GAL4; *ey*^3.5^-GAL4/*Tb, ey*^3.5^-GAL4/*Tb* and *Mz97*-GAL4; +/+ were either obtained from BDSC or generated in our previous studies [10, 11].

To knock down *mys* in both R cells and WG, genetic crosses were performed to generate flies with the genotype *Mz97*-GAL4/+; *ey*^3.5^-GAL4/*UAS-mys-RNAi-JF02819*. To knock down *scb* in eye-disc epithelium but not WG, genetic crosses were performed to generate flies with the genotype *ey*^3.5^*-*GAL4*/UAS-mys-RNAi-HMS00043*. To knock down *mys* in WG but not R cells, genetic crosses were performed to generate flies with the genotype *MZ97-*GAL4*/*+*; UAS-mys-RNAi-HMS00043/*+. To knock down *mys* specifically in R cells but not other cell types in the epithelial layer of the eye disc, genetic crosses were performed to generate flies with the genotype *C155-*GAL4*; UAS-mys-RNAi-HMS00043/*+.

To knock down *scb* in both R cells and WG, genetic crosses were performed to generate flies with the genotype *Mz97*-GAL4/+; *ey*^3.5^-GAL4/*UAS-scb-RNAi-JF02696*. To knock down *scb* in eye-disc epithelium but not WG, genetic crosses were performed to generate flies with the genotype *ey*^3.5^-GAL4/*UAS-scb-RNAi-JF02696*. To knock down *scb* specifically in WG but not R cells, genetic crosses were performed to generate flies with the genotype *Mz97*-GAL4/+; +/*UAS-scb-RNAi-JF02696*.

To knock down *rhea* in eye-disc epithelium but not WG, genetic crosses were performed to generate flies with the genotype *ey*^3.5^-GAL4/*UAS-rhea-RNAi-HMS00799* and *ey*^3.5^-GAL4/*UAS-rhea-RNAi-HMS00856*. To knock down *rhea* specifically in WG but not R cells, genetic crosses were performed to generate flies with the genotype *Mz97*-GAL4/+; +/*UAS-rhea-RNAi-HMS00799*. To generate large clones of eye mutant tissues homozygous for the strong *rhea* loss-of-function mutant allele *rhea*^1^, genetic crosses were performed to generate flies with the genotype *yw; P{GAL4-ey.H}3–8, P{UAS-FLP.D}JD1/*+*; P{white-un1}70C rhea*^*1*^ *P{neoFRT}80B/P{GMR-hid}SS3, l(3)CL-L1, P{Car20y}73C P{neoFRT}80B*.

### Histology

Eye–brain complexes from third-instar larvae were dissected and fixed for immunostaining similarly as described previously [10, 11].

### Immunostaining

Primary and secondary antibodies were used at following dilutions: mouse MAb24B10 (1:100; Developmental Studies Hybridoma Bank or DSHB Cat#24B10), mouse monoclonal anti-Mys (1:100; DSHB Cat#CF.6G11), mouse monoclonal anti-Repo (1:100; DSHB Cat#8D12), rabbit polyclonal anti-Bdl (1:500), rabbit polyclonal anti-HRP (1:500; Jackson ImmunoResearch Cat#323-005-021). Secondary antibodies including Alexa Fluor 488 goat anti-Mouse IgG (Invitrogen, Cat# A11001), Alexa Fluor 488 goat anti-Rabbit IgG (Invitrogen, Cat#A32731), Alexa Fluor 647 goat anti-Rabbit IgG (Invitrogen, Cat# A20991) and Alexa Fluor 647 goat anti-Mouse IgG (Invitrogen, Cat#A32728), were used at 1:500 dilution.

### Confocal microscopy

Epifluorescent images were captured and analyzed by confocal microscopy (Olympus FV1000). Photo stacks were performed using Z-stack projection by FluoView software.

### Statistical analysis

The number of sub-retinal glia in the eye disc of wild-type (n = 8) and *mys* knockdown (n = 8) third-instar larva was counted. For *mys* knockdown, only individuals showing the stalling phenotypes were included. Two-tailed Student’s t-tests were used for statistical analysis. The difference is considered as significant when a p value is < 0.05.

## Data Availability

The datasets supporting the conclusion of this study are included in this article.

## Abbreviations

Bdl: Borderless
BN: Bolwig’s Nerve
ECM: Extracellular matrix
ey: Eye disc
if: Inflated
la: Lamina
me: Medulla
mys: Myospheroid
ol: Optic lobe
os: Optic stalk
PG: Perineurial glia
R cells: Photoreceptor neurons
Repo: Reversed polarity protein
RNAi: RNA interference
Scb: Scab
Tutl: Turtle
WG: Wrapping glia

## References

[1] Stipursky J, Romao L, Tortelli V, Neto VM, Gomes FC (2011). Neuron-glia signaling: implications for astrocyte differentiation and synapse formation. Life Sci.

[2] He L, Lu QR (2013). Coordinated control of oligodendrocyte development by extrinsic and intrinsic signaling cues. Neurosci Bull.

[3] Altshuler D, Lillien L (1992). Control of photoreceptor development. Curr Opin Neurobiol.

[4] Sato M, Suzuki T, Nakai Y (2013). Waves of differentiation in the fly visual system. Dev Biol.

[5] Hadjieconomou D, Timofeev K, Salecker I (2011). A step-by-step guide to visual circuit assembly in Drosophila. Curr Opin Neurobiol.

[6] Chang YC, Tsao CK, Sun YH (2018). Temporal and spatial order of photoreceptor and glia projections into optic lobe in Drosophila. Sci Rep.

[7] Fernandes VM, Chen Z, Rossi AM, Zipfel J, Desplan C (2017). Glia relay differentiation cues to coordinate neuronal development in Drosophila. Science.

[8] Yuva-Aydemir Y, Klambt C (2011). Long-range signaling systems controlling glial migration in the Drosophila eye. Dev Neurobiol.

[9] Franzdottir SR, Engelen D, Yuva-Aydemir Y, Schmidt I, Aho A, Klambt C (2009). Switch in FGF signalling initiates glial differentiation in the Drosophila eye. Nature.

[10] Chen Y, Cameron S, Chang WT, Rao Y (2017). Turtle interacts with borderless in regulating glial extension and axon ensheathment. Mol Brain.

[11] Cameron S, Chen Y, Rao Y (2016). Borderless regulates glial extension and axon ensheathment. Dev Biol.

[12] Hansen M, Walmod PS (2013). IGSF9 family proteins. Neurochem Res.

[13] Rangarajan R, Gong Q, Gaul U (1999). Migration and function of glia in the developing Drosophila eye. Development.

[14] Hummel T, Attix S, Gunning D, Zipursky SL (2002). Temporal control of glial cell migration in the Drosophila eye requires gilgamesh, hedgehog, and eye specification genes. Neuron.

[15] Xie X, Gilbert M, Petley-Ragan L, Auld VJ (2014). Loss of focal adhesions in glia disrupts both glial and photoreceptor axon migration in the Drosophila visual system. Development.

[16] Liu Z, Chen Y, Rao Y (2020). An RNAi screen for secreted factors and cell-surface players in coordinating neuron and glia development in Drosophila. Mol Brain.

[17] Van Vactor D, Krantz DE, Reinke R, Zipursky SL (1988). Analysis of mutants in chaoptin, a photoreceptor cell-specific glycoprotein in Drosophila, reveals its role in cellular morphogenesis. Cell.

[18] Brower DL, Wilcox M, Piovant M, Smith RJ, Reger LA (1984). Related cell-surface antigens expressed with positional specificity in Drosophila imaginal discs. Proc Natl Acad Sci USA.

[19] Fernandes VM, McCormack K, Lewellyn L, Verheyen EM (2014). Integrins regulate apical constriction via microtubule stabilization in the Drosophila eye disc epithelium. Cell Rep.

[20] Alfonso TB, Jones BW (2002). gcm2 promotes glial cell differentiation and is required with glial cells missing for macrophage development in Drosophila. Dev Biol.

[21] Takada Y, Ye X, Simon S (2007). The integrins. Genome Biol.

[22] Brown NH, Gregory SL, Rickoll WL, Fessler LI, Prout M, White RA, Fristrom JW (2002). Talin is essential for integrin function in Drosophila. Dev Cell.

[23] Klapholz B, Brown NH (2017). Talin—the master of integrin adhesions. J Cell Sci.

[24] Martin KA, Poeck B, Roth H, Ebens AJ, Ballard LC, Zipursky SL (1995). Mutations disrupting neuronal connectivity in the Drosophila visual system. Neuron.

[25] Newsome TP, Asling B, Dickson BJ (2000). Analysis of Drosophila photoreceptor axon guidance in eye-specific mosaics. Development.

[26] Garrity PA, Rao Y, Salecker I, McGlade J, Pawson T, Zipursky SL (1996). Drosophila photoreceptor axon guidance and targeting requires the dreadlocks SH2/SH3 adapter protein. Cell.

[27] Deiner MS, Kennedy TE, Fazeli A, Serafini T, Tessier-Lavigne M, Sretavan DW (1997). Netrin-1 and DCC mediate axon guidance locally at the optic disc: loss of function leads to optic nerve hypoplasia. Neuron.

[28] Gong Q, Rangarajan R, Seeger M, Gaul U (1999). The netrin receptor frazzled is required in the target for establishment of retinal projections in the Drosophila visual system. Development.

[29] Tavares L, Pereira E, Correia A, Santos MA, Amaral N, Martins T, Relvas JB, Pereira PS (2015). Drosophila PS2 and PS3 integrins play distinct roles in retinal photoreceptors-glia interactions. Glia.

[30] Broadie K, Baumgartner S, Prokop A (2011). Extracellular matrix and its receptors in Drosophila neural development. Dev Neurobiol.

[31] Pasterkamp RJ, Peschon JJ, Spriggs MK, Kolodkin AL (2003). Semaphorin 7A promotes axon outgrowth through integrins and MAPKs. Nature.

[32] Wingerd KL, Goodman NL, Tresser JW, Smail MM, Leu ST, Rohan SJ, Pring JL, Jackson DY, Clegg DO (2002). Alpha 4 integrins and vascular cell adhesion molecule-1 play a role in sympathetic innervation of the heart. J Neurosci.

[33] Hines JH, Abu-Rub M, Henley JR (2010). Asymmetric endocytosis and remodeling of beta1-integrin adhesions during growth cone chemorepulsion by MAG. Nat Neurosci.

[34] Vogelezang MG, Liu Z, Relvas JB, Raivich G, Scherer SS (2001). ffrench-Constant C: Alpha4 integrin is expressed during peripheral nerve regeneration and enhances neurite outgrowth. J Neurosci.

[35] Hopker VH, Shewan D, Tessier-Lavigne M, Poo M, Holt C (1999). Growth-cone attraction to netrin-1 is converted to repulsion by laminin-1. Nature.

[36] Belkin AM, Stepp MA (2000). Integrins as receptors for laminins. Microsc Res Tech.

[37] Aumailley M (2013). The laminin family. Cell Adh Migr.

[38] Silies M, Yuva Y, Engelen D, Aho A, Stork T, Klambt C (2007). Glial cell migration in the eye disc. J Neurosci.

[39] Tsao CK, Huang YF, Sun YH (2020). Early lineage segregation of the retinal basal glia in the Drosophila eye disc. Sci Rep.

